# Colorectal cancer genomic biomarkers in the clinical management of patients with metastatic colorectal carcinoma

**DOI:** 10.37349/etat.2020.00004

**Published:** 2020-02-29

**Authors:** Anna Maria Rachiglio, Alessandra Sacco, Laura Forgione, Claudia Esposito, Nicoletta Chicchinelli, Nicola Normanno

**Affiliations:** Cell Biology and Biotherapy Unit, Istituto Nazionale Tumori-IRCCS-Fondazione G. Pascale, 80131 Naples, Italy; University of Southampton, UK

**Keywords:** Colorectal carcinoma, molecular biomarker, precision medicine

## Abstract

Colorectal carcinoma (CRC) is an heterogeneous disease in which different genetic alterations play a role in its pathogenesis and progression and offer potential for therapeutic intervention. The research on predictive biomarkers in metastatic CRC (mCRC) mainly focused on the identification of biomarkers of response or resistance to anti-epidermal growth factor receptor monoclonal antibodies. In this respect, international guidelines suggest testing mCRC patients only for *KRAS*, *NRAS* and *BRAF* mutations and for microsatellite instability. However, the use of novel testing methods is raising relevant issue related to these biomarkers, such as the presence of sub-clonal *RAS* mutations or the clinical interpretation of rare no-V600 *BRAF* variants. In addition, a number of novel biomarkers is emerging from recent studies including amplification of *ERBB2*, mutations in *ERBB2*, *MAP2K1* and *NF1* and rearrangements of *ALK*, *ROS1*, *NTRK* and *RET*. Mutations in *POLE* and the levels of tumor mutation burden also appear as possible biomarkers of response to immunotherapy in CRC. Finally, the consensus molecular subtypes classification of CRC based on gene expression profiling has prognostic and predictive implications. Integration of all these information will be likely necessary in the next future in order to improve precision/personalized medicine in mCRC patients.

## Introduction

Colorectal cancer (CRC) is the third most common cancer in men and the second most common in women [1]. Although screening programs have significantly improved the early diagnosis of CRC, a significant fraction of patients either have metastatic lesions at diagnosis or will develop metastases during follow up. As such, a significant part of cancer research has focused on identifying novel therapies in the field of metastatic CRC (mCRC) [2].

The availability of highly active chemotherapeutics and the approval of several targeted therapies, such as agents directed against the epidermal growth factor receptor (EGFR) and the vascular endothelial growth factor (VEGF) and its receptors, led to significant improvements in tumor response rates and survival in patients with mCRC [3]. However, despite the multitude of treatments available, outcomes and toxicity with each regimen can vary markedly from patient to patient [4]. The research on predictive biomarkers in mCRC mainly focused on the identification of biomarkers of response or resistance to anti-EGFR monoclonal antibodies [5]. However, CRC is a highly heterogeneous disease in which different signaling pathways contribute to cancer pathogenesis and progression [6]. Genetic alterations involved in the resistance to EGFR monoclonal antibodies might also offer opportunities for therapeutic intervention. Therefore, comprehensive genomic profiling might significantly improve the use of personalized therapy in patients with mCRC.

**Figure 1.**
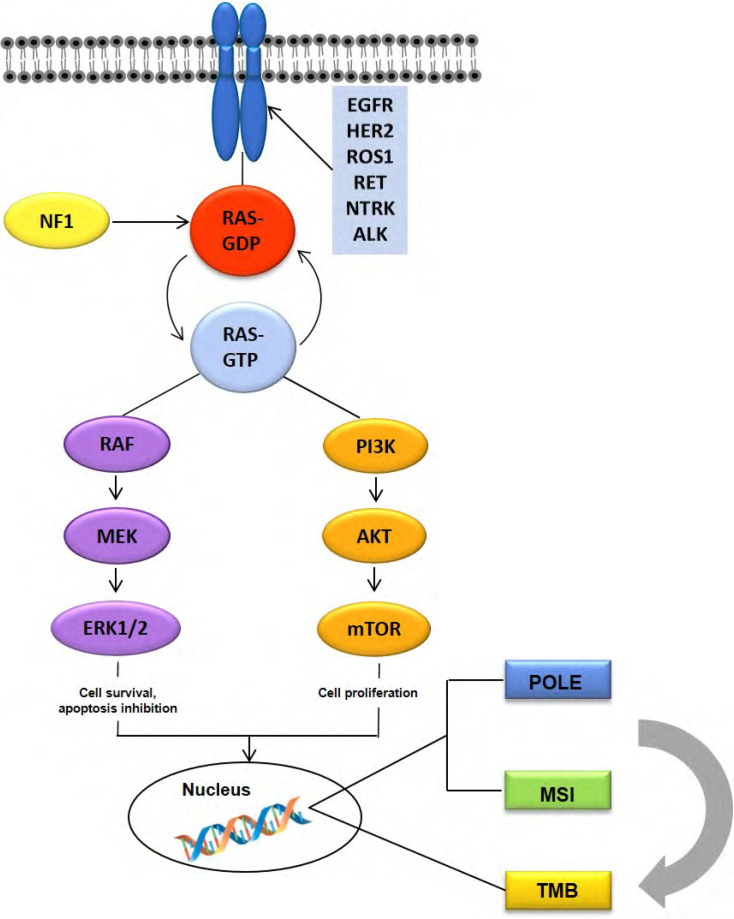
Genomic predictive or prognostic alterations in colorectal cancer

In this review article, we will briefly discuss the predictive biomarkers that have been currently approved by guidelines for the management of mCRC patients and their impact on treatment decision making, and we will next summarize current evidence on emerging biomarkers that might improve precision/personalized therapy in mCRC (Figure 1).

## Current Biomarkers

The guidelines of the European Society of Medical Oncology (ESMO) indicate as mandatory for the management of mCRC the testing of KRAS, NRAS and BRAF mutations and the assessment of the microsatellite instability (MSI) status [4] (Table 1).

**Table 1.** Summary of current and emerging molecular biomarkers associated with targeted therapies

**Biomarker**	**Agents**	**Status**
***KRAS/NRAS***	Cetuximab, Panitumumab (negative predictive biomarker)	Approved EMA and FDA
***BRAF***		
***V600E***	Encorafenib + Binimetinib + Cetuximab [57]	Approved FDA
***nonV600E***	MEK ± BRAF ± EGFR inhibitors	Experimental
**MSI**	Pembrolizumab [66, 67]; Nivolumab [68]	Approved FDA
**HER2**	Trastuzumab + Pertuzumab [84]; Lapatinib + Trastuzumab [85]	Experimental
***NTRK***	Larotrectinib, Entrectinib	Approved EMA (Larotrectinib) and FDA (Larotrectinib, Entrectinib)
***NF1***	MEK ± BRAF ± EGFR inhibitors	Experimental

### RAS

RAS is a family of GTPase proteins whose signaling regulates key processes in tumor pathogenesis, including cell proliferation, differentiation, adhesion, migration and survival [7]. Activating mutations of *RAS* genes are indeed involved in the progression of different tumor types. The three *RAS* genes (*HRAS*, *NRAS*, and *KRAS*) still represent the most mutated oncogene family in cancer (30%) and an estimated 52% of CRCs carry mutations in any *RAS* gene. *KRAS* is the most frequently mutated gene among the RAS family, representing 86% of *RAS* mutations in CRC, followed by 14% *NRAS* mutations [8]. *KRAS* mutations occur in approximately 44% of CRC, and the majority affect codons 12 (30%) and 13 (8%) of exon 2. An additional 6% of mutations are found in *KRAS* exons 3 and 4, while 5% of mutations are in *NRAS* exons 2, 3 or 4 [9].

RAS proteins are essential components of the EGFR signaling cascade and, therefore, their constitutive activation might lead to resistance to anti-EGFR monoclonal antibodies [5]. Indeed, a series of retrospective analyses has shown that *KRAS* exon 2 mutations (codons 12 and 13) are associated with resistance to anti-EGFR therapy in patients with mCRC, in the context of randomized studies in which the anti-EGFR cetuximab or panitumumab monoclonal antibodies were used alone or in combination with chemotherapy [10–17] (Table 2). Subsequently, activating *KRAS* hotspot mutations in exons 3 and 4 and in exons 2, 3 and 4 of *NRAS* were found to also predict for lack of benefit from anti-EGFR therapies, refining the population of patients who might benefit these agents [18–23] (Table 2).

**Table 2.** Main clinical trials that explored predictive biomarkers in CRC

**Biomarker**	**Clinical trial**	**Results**
***RAS***	PRIME [15]	The combination of panitumumab and FOLFOX4 significantly improved PFS in patients with KRAS wild-type tumors.
	CRYSTAL [20]	Molecular testing of tumors for all activating RAS mutations is essential before considering anti-epidermal growth factor receptor therapy, thereby allowing the further tailoring of cetuximab administration to maximize patient benefit.
	OPUS [16]	The addition of cetuximab to FOLFOX-4 significantly improved PFS and response in patients with KRAS wild-type tumors.
	MRC COIN [17]	This trial has not confirmed a benefit of addition of cetuximab to oxaliplatin-based chemotherapy in first-line treatment of patients with advanced CRC.
	FIRE-3 [23]	In this study a significantly higher OS was demonstrated for FOLFIRI plus cetuximab compared to FOLFIRI plus bevacizumab in the extended RAS wild-type subgroup.
	NCT03600883 [35]	Early results suggest antitumor activity of single-agent AMG 510 in KRAS^G12C^ mutant solid tumors.
	NCT03785249 [33]	The trial is ongoing and responses to treatment with MRTX849 have been observed in both lung and CRC KRASG12C mutant patients.
***BRAF***	SWOG 1406 [62]	The addition of vemurafenib to the combination of cetuximab and irinotecan in *BRAF*V600 mutated tumors resulted in a prolongation of PFS and a higher disease control rate.
	BEACON CRC [63]	A combination of encorafenib, cetuximab, and binimetinib resulted in significantly longer OS and a higher response rate than standard therapy in patients with mCRC with the BRAF V600E mutation.
**MSI**	NCT01876511 [71]	This study showed that mismatch-repair status predicted clinical benefit of immune checkpoint blockade with pembrolizumab.
	CheckMate 142 [73]	Nivolumab provided durable responses and disease control in pre-treated patients with dMMR/MSI-H mCRC.
**HER-2**	HERACLES [81]	The combination of trastuzumab and lapatinib is active and well tolerated in treatment-refractory patients with HER2-positive tumors.
	MyPathway [86]	Durable responses were seen in patients with refractory colorectal cancers with HER2 activation/overexpression when the approved targeted therapy regimen administered without chemotherapy.
**CMSs**	CALGB/SWOG 80405 [131]	The CMSs are highly prognostic and predictive for OS and PFS.
	Fire-3(AIO KRK-0306) [132]	Prolonged OS induced by FOLFIRI plus cetuximab versus FOLFIRI plus bevacizumab in the FIRE-3 study appears to be driven by CMS2 and CMS4.

OS: overall survival

Testing for *RAS* mutational status is currently recommended for all patients at the time of mCRC diagnosis [4, 24]. The introduction in clinical molecular diagnostics of novel techniques with high sensitivity raised the question of the threshold of *RAS* mutations to identify patients that are truly resistant to anti-EGFR agents. Although *RAS* mutations are an early event in CRC pathogenesis in most cases, a fraction of CRC carrying sub-clonal *RAS* mutations has been identified [25, 26]. The occurrence of sub-clonal *KRAS* mutations at low allelic frequency has been confirmed in studies that explored the use of liquid biopsy for *KRAS* mutation testing in mCRC [27, 28]. Recent publications suggested that a 5% cutoff should be the optimal threshold for the detection of *RAS* mutations in the clinical scenario [29, 30].

Finally, *RAS* mutations have been investigated in CRC only as marker of resistance to anti-EGFR agents up to now because of the lack of active agents targeting RAS proteins. However, promising preliminary results have been obtained with novel drugs targeting *RAS* mutations [31]. These findings are opening new therapeutic opportunities for patients carrying *RAS* mutant CRC (Table 2).

In particular, different drugs targeting the KRASG12C mutation are in clinical development in patients with solid tumors, including CRC. In a recent study, Hallin et al. [32], showed that MRTX849 is a selective and covalent KRASG12C inhibitor. Indeed, this small molecule caused tumor shrinkage in 65% of KRASG12C positive cell line-derived and patient-derived xenograft models of multiple solid tumor types. Furthermore, objective responses have been observed in KRASG12C-positive lung and colon adenocarcinoma patients treated with MRTX849 [32]. In an ongoing phase I/II trial responses to this drug have been observed in both lung and CRC KRASG12C mutant patients [33] (Table 2).

Another novel small molecule that specifically and irreversibly inhibits KRASG12C by locking it in an inactive GDP-bound state is AMG 510 [34]. A phase 1 open-label, multicenter study is underway to evaluate the safety, tolerability, pharmacokinetics, and efficacy of AMG 510 in patients with locally-advanced/ metastatic KRASG12C solid tumors and the preliminary data suggest the antitumor-activity of this agent in different tumor types, including CRC [35] (Table 2).

### BRAF

Approximately 8% of advanced CRC cases carry activating mutations of *BRAF*, a key protein in the mitogen-activated protein-kinase (MAPK) signaling pathway [36, 37]. Roughly 90% of *BRAF* mutations in CRC occur as a T1799 transversion in exon 15, which leads to the substitution of valine for glutamic acid (V600E) [38]. This substitution regulates phosphorylation, increasing BRAF activity by approximately ten times compared to the wild-type protein [39]. Of note, patients carrying *BRAF* V600E mutations have distinct clinical and pathological features including right sided tumors, high grade, older age, female sex, T4 tumors, mucinous histology, poorly differentiated tumors and microsatellite instability [40–42]. Nevertheless, *BRAF* mutations can also be detected in left-sided tumors [43].

The *BRAF* V600E mutation is a strong negative prognostic biomarker in mCRC, as demonstrated in several clinical trials and metanalyses [13, 21, 44–46]. In patients with disease recurrence after primary tumor resection, the presence of the *BRAF* V600E mutation is associated with reduced post-recurrence survival [47]. Similarly, some studies have shown that in patients receiving liver metastasis resection, the *BRAF* V600E mutation is correlated with shorter survival [48–50]. However, approximately 25% of patients with *BRAF* V600E mutations have a good outcome, similar to no-*BRAF* mutant cases, thus suggesting that the *BRAF* V600E population of CRC patients is heterogeneous [51]. The *BRAF* V600 mutations are classified as class I mutations leading to RAS-independent high level of activation of BRAF, based on functional studies on non-CRC preclinical models [52, 53]. In contrast, class II mutations (affecting codons 597 and 601) induce intermerdiate, RAS-independent BRAF activation, while class III variants (in codons 594 and 596) are RAS-dependent and produce a low level of BRAF activation. Alterations in class II or III are less frequent as compared to class I and account for 2.2% of all patients tested or 21.6% of all *BRAF* mutations in CRC [54]. Recent data suggest that class III *BRAF* mutations are associated with a favorable prognosis in patients with mCRC, while class II mutant cases seem to have an outcome similar to patients with class I *BRAF* mutations [54–56]. However, the limited number of cases included in these studies suggests that additional experimentation should be performed in this field.

While the negative prognostic role of the *BRAF* V600E mutations is quite clear, its predictive role as a mechanism of resistance to anti-EGFR monoclonal antibodies-based therapy in the first-line treatment of mCRC patients is less established. In particular, two meta-analyses on the predictive role of the *BRAF* V600E mutation for anti-EGFR agents came to completely different conclusions, although analyzing the same clinical trials. Pietrantonio et al. [57], concluded that the *BRAF* V600E mutations are a negative predictive factor for anti-EGFR agents, thus supporting the exclusion of patients carrying such genetic alterations from treatment with these agents. In contrast, another meta-analysis by Rowland et al. [58], concluded that the evidence is insufficient in order to justify the exclusion of anti-EGFR agents in the case of patients with *BRAF* V600E mutations. Importantly, pre-clinical data suggest that patients with class III mutations may be sensitive to EGFR inhibitors [53].

The poor prognosis of *BRAF* V600 mutant mCRC patients led to the exploration of novel therapeutic approaches. Unlike what occurrs in melanoma, mCRC patients with *BRAF* V600E mutation do not respond to BRAF inhibitors. This phenomenon is due to a specific molecular mechanism demonstrated in CRC cells that involves the activation of alternative signal transduction pathways through the EGFR [59]. This last observation led to explore the activity of drug combinations containing BRAF, MEK and/or PI3K inhibitors together with anti-EGFR monoclonal antibodies. An open-label phase I/II study in patients with *BRAF* V600E mutation-positive mCRC, demonstrated that patients receiving triple therapy (dabrafenib, trametinib and panitumumab) had a numerically improved objective response rate (ORR) (21%) compared with those receiving panitumumab plus either dabrafenib (10%) or trametinib (0%) and had a longer progression free survival (PFS) (4.2 *vs* 3.5 *vs* 2.6 months) [60, 61]. Moreover, in a randomized trial, addition of vemurafenib to the combination of cetuximab and irinotecan resulted in prolonged PFS [4.4 *vs* 2.0 months, hazard ratio (HR) 0.42, 95% confidence interval (CI) 0.26–0.66, *P* < 0.001] and a higher disease control rate (67% *vs* 22%; *P* < 0.001) compared with cetuximab and irinotecan treatment alone in heavily pre-treated patients with BRAF-mutant mCRC [62] (Table 2).

The combination of encorafenib (BRAF inhibitor), binimetinib (MEK inhibitor) and cetuximab is being assessed in the BEACON trial in *BRAF*-mutant mCRC patients who had disease progression after one or two previous regimens. Patients are randomly assigned to receive encorafenib, binimetinib, and cetuximab (triplet-therapy group); encorafenib and cetuximab (doublet-therapy group); or the investigators’ choice of either cetuximab and irinotecan or cetuximab and FOLFIRI (control group). The results of a pre-specified interim analysis of the BEACON trial showed a median overall survival of 9.0 months in the triplet-therapy group, 8.4 months in the doublet-therapy group and 5.4 months in the control group [63] (Table 2). Based on these data, the Food and Drug Administration (FDA) approved such combination for *BRAF* V600 mutant mCRC patients (Table 1).

### Microsatellite instability

Microsatellites are repeated DNA sequences of 1–6 bp that can be found in coding and noncoding regions of the genome. Microsatellite instability (MSI) results from inactivation of the mismatch repair (MMR) genes through sporadic *MLH1* promoter hypermethylation and/or germline/somatic mutations in MMR genes, such as *MLH1*, *MSH2*, *MSH6*, or *PMS2* that are also involved in the Lynch syndrome [64, 65]. MMR deficiency leads to the accumulation of somatic mutations and induces genomic instability, causing cancer-associated alterations [66]. MSI is detected in approximately 15% of patients with mCRC; only 3% of the cases are associated with Lynch syndrome and the other 12% are caused by sporadic hyper-methylation of the *MLH1* gene and/or germline/somatic mutations in MMR genes [67].

MMR/MSI status testing is recommended for patients with CRC for prognostic stratification [24]. Based on the number of markers altered, CRC are classified in MSI-high (MSI-H), MSI-low (MSI-L) and microsatellite stable (MSS) if the test is performed on DNA, and in MMR-proficient (pMMR) or deficient (dMMR) if the MMR proteins are tested with immunohistochemistry. In clinical practice, the recommended method for MSI/MMR testing is immunohistochemistry. Alternatively, testing can be performed by polymerase chain reaction (PCR)-based assessment of microsatellite alterations using five microsatellite markers, including at least BAT-25 and BAT-26. Finally, next-generation sequencing (NGS), coupling MSI and tumor mutation burden (TMB) analysis with comprehensive genomic profiling, may represent a more adequate tool for selecting patients for immunotherapy, for common or rare cancers not belonging to the spectrum of Lynch syndrome [68].

A number of different studies have demonstrated that early stage MSI-H CRC patients have better prognosis as compared with MSI-L and MSS patients [69]. As a consequence of the good prognosis of MSI-H tumors, this biomarker is positive in only about 4% of mCRC. However, the prognostic significance of MSI in the advanced stages of the disease is different from the initial ones. In fact, in mCRC the presence of MSI is associated with a worse prognosis as compared with MSS [44].

MMR deficient tumors have a high mutational load that can produce multiple immunogenic neoantigens, which will increase the probability of response to immune checkpoint inhibitors [70]. In this regard, several phase II clinical trials demonstrated a high rate of responses to PD-1/PD-L1 inhibitors in patients with mCRC and MSI-H or dMMR [71–73] (Table 2). Interestingly, responses have been obtained in MSI patients with different histological types of cancer [72]. Based on these data, the FDA with a historic decision approved the use of pembrolizumab in patients with MSI regardless of the histological origin of the tumor (Table 1).

Different studies confirmed that MSI CRC has a higher TMB as compared with MMS tumors [74, 75]. In particular, a very good correlation between MSI-status and TMB levels was found in CRC [64]. However, a wide range of TMB value have been found also among MSI CRC. In this respect, patients with MSI CRC and high TMB showed and higher ORR and a longer PFS and OS as compared with MSI CRC patients with lower TMB. Therefore, TMB appears to be an important independent biomarker within MSI-H mCRC to stratify patients for likelihood of response to immune checkpoint inhibitors [75].

## Emerging biomarkers

### HER2 amplification and mutations

The HER2 oncogene is coded by the *ERBB2* gene and belongs to the EGFR family of tyrosine kinase receptors that includes also EGFR, HER-3 and HER-4.

Recent studies have highlighted a possible role of genetic alterations of HER2 in the pathogenesis of CRC. Amplification or mutations of *ERBB2*, both point and small insertions/deletions, have been described in a subset of patients with CRC [6]. The percentage of cases with *ERBB2* alterations varies considerably between studies, probably due to the limited number of cases analyzed and the variety of analysis methods used, especially for gene amplification studied in some cases only at the level of protein expression by immunohistochemistry [76]. A recent study, which analyzed 8,887 cases of mCRC by means of NGS techniques, highlighted the presence of *ERBB2* amplification in 2.8% of cases, mutations in 1.5% and amplification and mutation in 0.4% [77]. Since the frequency of *RAS* mutations is generally lower among cases with *ERBB2* amplification than in those without amplification, the frequency of *ERBB2* amplification in wild-type *RAS* tumors is expected in the order of 4–5% [76, 77]. Overexpression of HER2 correlates in CRC with an aggressive tumor behavior, which includes profound invasion, lymphatic metastases, distant metastases, perineural invasion [78–80]. However, there are conflicting data on the prognostic role of *ERBB2* alterations as well as on the association with the disease site [76].

Some studies have explored the predictive role of *ERBB2* amplification as biomarker of resistance to anti-EGFR drugs in mCRC patients [81–85]. These studies described a reduced response rate and a shorter survival in mCRC patients with *ERBB2* amplification compared to those without *ERBB2* amplification when treated with anti-EGFR monoclonal antibodies. However, these studies are all retrospective analyses, with heterogeneous cases treated in different lines and with different drug combinations. More importantly, all patients included in these analyses (negative and positive for the amplification of *ERBB2*) received treatment with anti-EGFR drugs. Therefore, these studies do not allow formally distinguishing between a prognostic or predictive effect of *ERBB2* amplification, which should be explored in the context of randomized trials. Finally, the predictive value of *ERBB2* amplification may be different in the various treatment lines. Sartore-Bianchi et al. [85], have described a trend of different HR between the I, II and III–IV treatment line for the predictive value of *ERBB2* amplification in mCRC patients treated with anti-EGFR drugs, although not statistically significant.

Encouraging preliminary data suggest that *ERBB2* amplification could be an important marker of response to anti-ERBB2 drugs. In phase II studies that used different combinations of anti-ERBB2 drugs, response rates ranging between 30% and 71% have been described [81, 86–89] (Table 2). In particular, the phase 2 HERACLES study evaluated the efficacy of trastuzumab and lapatinib in 27 HER2-positive/*KRAS* exon 2 wild-type patients with mCRC who had progressed on all other therapies, including cetuximab [81]. An ORR of 30% was observed, with additional 44% of the patients experiencing a stabilization of the disease. The mPFS was 21 weeks. However, the mPFS of patients harboring tumors with high levels of *ERBB2* gene copy number (≥ 945 as assessed by real time PCR) was 29 weeks, compared with a mPFS of 16 weeks for patients with tumors scoring below this threshold. These data suggest that a quantitative assessment of *ERBB2* amplification is needed to identify patients that are more sensitive to anti-HER2 drugs. In agreement with this hypothesis, a correlation was found between plasma copy number of *ERBB2* and activity of trastuzumab plus lapatinib in mCRC patients [90]. In the phase 2 basket study MyPathway, 57 mCRC patients with *ERBB2* amplification were treated with trastuzumab plus pertuzumab [89]. One (2%) patient had a complete response and 17 (30%) had partial responses, with an ORR of 32%. In this heavily pre-treated cohort of patients (a median of four previous treatment regimens), mPFS was 2·9 months and estimated middle overall survival (mOS) was 11·5 months. An exploratory analysis revealed that 13/56 tumors tested had cooccurring *KRAS* mutations. In a post-hoc exploratory analysis of survival outcomes by *KRAS* mutation status, ORR, mPFS and mOS were notably worse in patients with *KRAS*-mutated tumors as compared with those with *KRAS* wild-type disease. Numerous studies are underway and their results will be important to better define the role of anti-ERBB2 therapies in mCRC [86, 91].

A pre-clinical study suggested that CRC cells carrying activating mutations of *ERBB2* are sensitive to anti-HER2 agents [92]. However, the results of a basket trial in which patients with *ERBB2* mutations were treated with the pan-HER inhibitor neratinib showed that mCRC patients did not respond to this therapy unlike other histological types, although carrying similar mutations [93]. While these data must be considered preliminary, this observation raises the question whether *ERBB2* mutations are true drivers of tumor growth in CRC (Table 1).

### PIK3CA

The *PIK3CA* gene encodes the p110α subunit of the phosphatidylinositol-3-kinases (PI3K), which is activated by growth factor stimulation through receptor tyrosine kinases and in turn activates the AKTmTOR signaling pathway [94]. When mutated, PIK3CA induces constitutive phosphorylation of AKT, which promotes cell growth and suppresses apoptosis in cancer cells. [95].

The *PIK3CA* gene is mutated in many different tumors, including CRC [94]. In particular, mutations of *PIK3CA* have been described in 15–20% of CRC, with about 80% of mutations found in exons 9 and 20 [96–98]. In CRC, *PIK3CA* mutations are frequently associated with other molecular alterations, including *KRAS* mutations (especially exon 9 mutations) and high-degree CpG island methylator phenotype [99].

While *PIK3CA* mutations seem not to have a prognostic role in CRC [100], their predictive role in both early and metastatic CRC has been investigated in several studies.

In patients with early CRC, the presence of *PIK3CA* mutations was associated with a reduced risk of relapse in patients with regular use of aspirin [101]. In particular, in patients with *PIK3CA* mutated CRC, the use of aspirin was associated with a reduction of tumor specific and overall mortality of 82% and 46%, respectively. This effect could be due to the ability of *PIK3CA* to induce the expression of cyclooxygenase-2 (COX2). However, rofecoxib, a selective COX2 inhibitor, did not show any effect on tumor recurrence in *PIK3CA* mutant CRC patients, suggesting that other, non-COX2-related mechanisms of action of aspirin might play a relevant role in its protective effect [102].

Several retrospective studies investigated the impact of *PIK3CA* mutations on the outcome of mCRC receiving anti-EGFR monoclonal antibodies. While some studies suggested a possible role of *PIK3CA* mutations in the primary resistance to anti-EGFR agents [103–107], other studies failed to confirm such correlation [108].

The discordance between these studies could be due in part to the different mechanisms of action of the exon 9 and exon 20 *PIK3CA* mutations. Exon 9 mutations interfere with the inhibitory activity of the p85 regulatory subunit and, therefore, require interaction with RAS proteins for activation. In contrast, mutations in exon 20 lead to constitutive activation of PI3K enzymatic activity independently from RAS [94]. In agreement with this hypothesis, in a retrospective study, including 743 mCRC patients receiving anti-EGFR therapies, De Roock et al. [98], found that in *KRAS* wild-type tumors, *PIK3CA* exon 9 mutations had no effect, whereas exon 20 mutations were associated with poor clinical response to cetuximab compared to wild-type PIK3CA tumors. It must be emphasized that exon 20 *PIK3CA* mutations are relatively rare in mCRC (2–3% of the cases) and this makes difficult to confirm their predictive role within randomized clinical trials.

### Gene fusions

*ALK*, *ROS1*, *NTRK* and *RET* rearrangements have been described in CRC [109–113]. Although these genetic alterations are present in CRC at a quite low frequency (< 1%), they seem more frequent in CRC patients with microsatellite instability.

Gene fusions can represent important targets for therapeutic intervention. In particular, NTRK inhibitors have demonstrated clinical activity in patients with gene rearrangements that result in fusions of *NTRK 1*, *2* and *3* with different partners [114]. The activity of NTRK inhibitors has been demonstrated in patients with *NTRK* fusions regardless of the histological type and the FDA and the European Medicine Agency have subsequently approved for the first time *NTRK* fusions as an agnostic marker (independent of histology) for a target therapy (Table 1).

### NF1

The *NF1* gene encodes for neurofibromin 1, which functions as a negative regulator of KRAS [115]. Previous studies demonstrated that inactivation of NF1 leads to resistance to EGFR tyrosine kinase inhibitors in lung cancer [116]. In a recent study of cetuximab-based therapy in a small cohort of Chinese mCRC patients, *NF1* mutations were associated with the shortest PFS [117]. In agreement with these findings, analyses of cohorts of mCRC patients who did not benefit from cetuximab treatment identified *NF1* mutations as possible mechanisms of intrinsic resistance to anti-EGFR monoclonal antibodies [118, 119]. Functional studies in CRC cells in which the *NF1* gene was inactivated confirmed the potential role in resistance to anti-EGFR monoclonal antibodies. Further studies are definitely needed to confirm these preliminary findings (Table 1).

### MAP2K1

Mutations of the *MAP2K1* gene coding for MEK1 have been consistently found to be associated with de novo and acquired resistance to anti-EGFR agents in different studies [118–121]. Mechanistically, these variants lead to constitutive activation of MEK1 and increased downstream signaling. Although activating mutations of the catalytic site of MEK1 are quite rare in CRC, their identification might lead to a better identification of patients with primary resistance to anti-EGFR monoclonal antibodies.

### POLE

The *POLE* gene encodes for the proofreading exonuclease domain of polymerase epsilon [122]. Pathogenic somatic *POLE* mutations occur in approximately 1.0% of CRCs. The patients who harbor these mutations have hypermutated tumors, which are expected to carry increased neoantigen load that may predict a response to immunotherapy [6, 123]. In a retrospective analysis of more than 4,500 patients with stage II/ III CRC, the presence of *POLE* mutations identified a subset of CRC patients with favorable prognosis [123]. Due to the very good prognosis of *POLE* mutant CRC, we might expect that a very limited fraction of mCRC patients carry mutations in *POLE*.

### Consensus molecular subtypes

Different classification of CRC based on gene expression profiling have been proposed [124–128]. In order to solve inconsistencies among the reported gene expression-based classifications and facilitate clinical translation, the International Colorectal Cancer Consortium proposed a classification of CRC into four distinct consensus molecular subtypes (CMS) [129]. CMS-1, the MSI immune, represents 14% of all CRC and shows higher rates of MSI, CpG island methylator phenotype high, hypermutation, and *BRAF* mutations. The CMS-2, canonical subtype, is found in 37% of CRC, and is associated with higher rates of somatic copy number alterations and WNT and MYC pathways activation. The CMS-3 metabolic subtype represents 13% of CRC. This group is characterized by lower rates of copy number alterations, high rates of *KRAS* mutations and metabolic dysregulation. The CMS-4 mesenchymal subtype including 23% of CRC shows high rates of transforming growth factor-β activation and angiogenesis. Finally, 13% of CRC cases have mixed features and they possibly represent a transition phenotype or intra-tumor heterogeneity [129].

The CMS classification has a prognostic value. In fact, the CMS-4 CRC group has the worse OS [129, 130]. However, the CMS-1 subtype shows the worse survival after relapse. This observation is consistent with the poor outcome of MSI-H mCRC patients.

The CALGB/SWOG 80405 and FIRE-3 studies explored the correlation between CMS classification and activity of cetuximab- and bevacizumab-based first line therapy in mCRC patients [131, 132] (Table 2). Quite surprisingly, the two studies reported quite different conclusions. While in the CALGB/SWOG 80405 trial, cetuximab treatment was correlated with a longer OS as compared with bevacizumab in the CMS-2 and CMS-3 subgroups, in the FIRE 3 study cetuximab did better than becvacizumab in all subgroups but CMS-3. Several factors, including the different chemotherapy backbone of the two trials and the use of different methods for CMS classification, might have caused such discrepancy [133]. Other studies confirmed that the CMS-2 CRC subgroup is likely the most sensitive to anti-EGFR agents [119]. However, patients in subgroups other than CMS-2 might respond to anti-EGFR monoclonal antibodies, and some CMS-2 CRC might be refractory to anti-EGFR based-therapy [119]. Taken together, these findings suggest that the predictive value of the CMS classification needs further evaluation in prospective clinical trials.

## Conclusions and future perspectives

While international guidelines suggest testing mCRC patients for *KRAS*, *NRAS*, *BRAF* and MSI only, the evidence presented in this review suggests that many other biomarkers may have an important role in personalizing treatments in mCRC. In particular, some genetic alterations may represent mechanisms of primary resistance to anti-EGFR agents but above all offer the possibility of targeted therapy. For example, tumors carrying genetic alterations in the MAPK pathway such as *MAP2K1* or *NF1* mutations might benefit treatment with combinations of MEK, BRAF and/or anti-EGFR agents (Table 1). A consideration that needs to be made is that many of the alterations discussed are relatively rare in mCRC. This poses a problem in the clinical validation of their prognostic role, which should be performed in randomized clinical trials. One possible solution could be to group all the alterations in a single panel, as recently proposed [134]. However, we must admit that each genetic alteration could have a different biological role.

The classification of CRC based on its driver mutations revealed the presence of a remarkable intertumor heterogeneity. To this phenomenon, however, is added that of intra-tumor heterogeneity. Numerous studies have shown that tumors, including CRC, often contain several clones. In this regard, multiple driver mutations, sometime at different allelic frequencies, are often identified in CRC [107]. Low-frequency allelic mutations may play a role in primary or acquired resistance to targeted therapies. For example, *RAS* mutations at low allelic frequency have been detected in some CRC patients who demonstrated resistance to anti-EGFR drugs [27, 28, 134]. It is possible that these mutations are selected during the neoplastic progression and the treatments received by the patient. Indeed, evidence suggests that tumor heterogeneity increases significantly during treatment, favoring the appearance of resistant clones. In this regard, the majority of the mechanisms involved in primary resistance to anti-EGFR drugs play also a role in acquired resistance. On the other hand, genetic alterations at low allelic frequency might represent sub-optimal targets for therapeutic intervention. Therefore, information on the clonality of the identified mutations should be included in the referral and discussed in the molecular tumor board for treatment decision.

In this scenario, liquid biopsy and in particular the testing of circulating cell-free DNA (cfDNA) might allow to better recapitulate the heterogeneity of mCRC and provide relevant information for the clinical management of CRC patients. Indeed, we and other groups found that cfDNA testing allows to identify RAS mutations that are at low allelic frequency in the primary tumors but that are possibly enriched in metastatic sites [27, 28]. Tracking genetic alterations in the cfDNA might also allow monitoring the response to treatment and the molecular evolution of the disease [135]. The development of high sensitive NGS-based methods for cfDNA testing is expanding the use of this approach also to early diagnosis and detection of minimal residual disease.

In the age of immunological therapy, many efforts have been made to optimize this approach in CRC with hitherto disappointing results overall. With the exception of MSI tumors, the majority of CRC presents characteristics of immune desert or immune tolerance. Novel approaches are definitely required in this context. In this respect, it is important to underline that also MSI tumors seem to be an heterogeneous group of tumors with some case showing an immunological background similar to MSS CRC [136].

The progress of precision medicine in the CRC requires some fundamental steps. First, it appears necessary to carry out an overall genomic and molecular profiling of CRC, which takes into account not only the genetic alterations but also the gene expression profiles and the characteristics of the tumor microenvironment. Furthermore, it will be necessary to develop algorithms to integrate all this information in order to identify the best therapeutic strategy for each individual patient. Only this approach will guarantee the application of precision medicine to the majority of patients with CRC.

## Abbreviations

cfDNA: circulating cell-free DNA
CI: confidence interval
CMS: consensus molecular subtypes
COX2: cyclooxygenase-2
CRC: colorectal cancer
dMMR: mismatch repair-deficient
EGFR: epidermal growth factor receptor
ESMO: European Society of Medical Oncology
FDA: Food and Drug Administration
HR: hazard ratio
MAPK: mitogen-activated protein-kinase
mCRC: metastatic colorectal cancer
MMR: mismatch repair
mOS: middle overall survival
MSI: microsatellite instability
MSI-H: microsatellite instability-high
MSI-L: microsatellite instability-low
NGS: next-generation sequencing
ORR: objective response rate
OS: overall survival
PCR: polymerase chain reaction
PFS: progression free survival
PI3K: phosphatidylinositol-3-kinases
pMMR: mismatch repair-proficient
TMB: tumor mutation burden
VEGF: vascular endothelial growth factor

## Declarations

### Copyright

© The Author(s) 2020.
